# Ankylosing spondylitis associated with Sweet’s syndrome: a case report

**DOI:** 10.1186/1752-1947-7-16

**Published:** 2013-01-10

**Authors:** Samia Mansouri, Fatima Ezzahra Abourazzak, Nassira Aradoini, Asmae Bettioui, Maryam Fourtassi, Latifa Tahiri, Fatima Zahra Mernissi, Siham Tizniti, Taoufik Harzy

**Affiliations:** 1 Rheumatology Department, Hassan II University Hospital, Fez, Morocco; 2 Dermatology Department, Hassan II University Hospital, Fez, Morocco; 3 Physical Medicine and Rehabilitation Department, Hassan II University Hospital, Fez, Morocco; 4 Radiology Department, Hassan II University Hospital, Fez, Morocco

**Keywords:** Ankylosing spondylitis, Sweet’s syndrome

## Abstract

**Introduction:**

Sweet’s syndrome is an acute neutrophilic dermatosis characterized by a diffuse dermal infiltrate of mature neutrophils. In most cases, it occurs as an isolated phenomenon (idiopathic Sweet’s syndrome) but it can be drug induced or associated with a variety of underlying diseases such as infections, neoplasms, and chronic inflammatory diseases. The association between Sweet’s syndrome and ankylosing spondylitis is rare. Only a few cases have been reported in the literature. We report a new case in which we describe an outbreak of acute neutrophilic dermatosis revealing ankylosing spondylitis.

**Case presentation:**

A 33-year-old Moroccan man presented with large-joint polyarthralgia, inflammatory pain in his buttocks and lower lumbar spine, fever and skin lesions. On examination, the patient had a low-grade fever, six tender but not swollen joints, limitation of motion of the lumbar spine, and painful erythematous maculopapules over his face, neck, and hands. Laboratory tests showed hyperleukocytosis, and elevated erythrocyte sedimentation rate and C-reactive protein. The immunological tests and infectious disease markers were negative. Investigations for an underlying neoplastic disease remained negative. Magnetic resonance imaging showed a bilateral sacroiliitis. Skin biopsy findings were consistent with Sweet’s syndrome. The diagnosis of Sweet’s syndrome associated with ankylosing spondylitis was established. Nonsteroid anti-inflammatory drugs were started and the patient showed rapid clinical and biological improvement.

**Conclusion:**

Three observations of the association between Sweet’s syndrome and spondylarthropathy have been reported in the literature. The cause of this association remains unclear. Some hypotheses have been developed, but further studies are needed to confirm or refute them.

## Introduction

Sweet’s syndrome (SwS) is a rare disease characterized by a constellation of clinical symptoms, physical features, and pathologic findings which include fever, neutrophilia, tender erythematous skin lesions (papules, nodules, and plaques), and a diffuse infiltrate consisting predominantly of mature neutrophils [1]. It was originally described by Dr Robert Douglas Sweet in the August-September 1964 issue of the *British Journal of Dermatology* as an “acute febrile neutrophilic dermatosis” [2]. SwS usually presents as an idiopathic disease (2 out of 3 of cases), but can also be drug induced or associated with other disorders such as infections, malignancies, and inflammatory conditions like chronic rheumatic diseases. The association between SwS and spondylarthropathy is rare. To the best of our knowledge, only three cases of SwS associated with spondylarthropathy have been reported in the literature [3-5]. We report a new case of SwS revealing ankylosing spondylitis (AS).

## Case presentation

**Figure 1:**
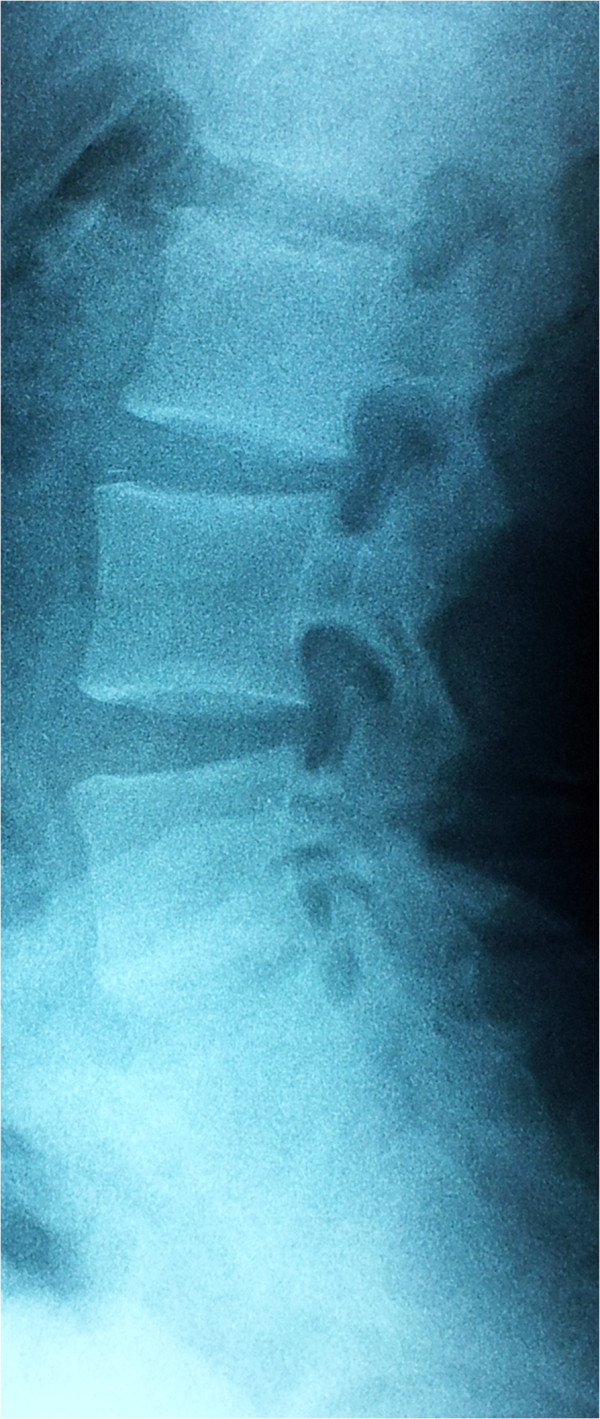
Plain X-ray of lumbar spine showing squaring of the lumbar vertebrae.

**Figure 2:**
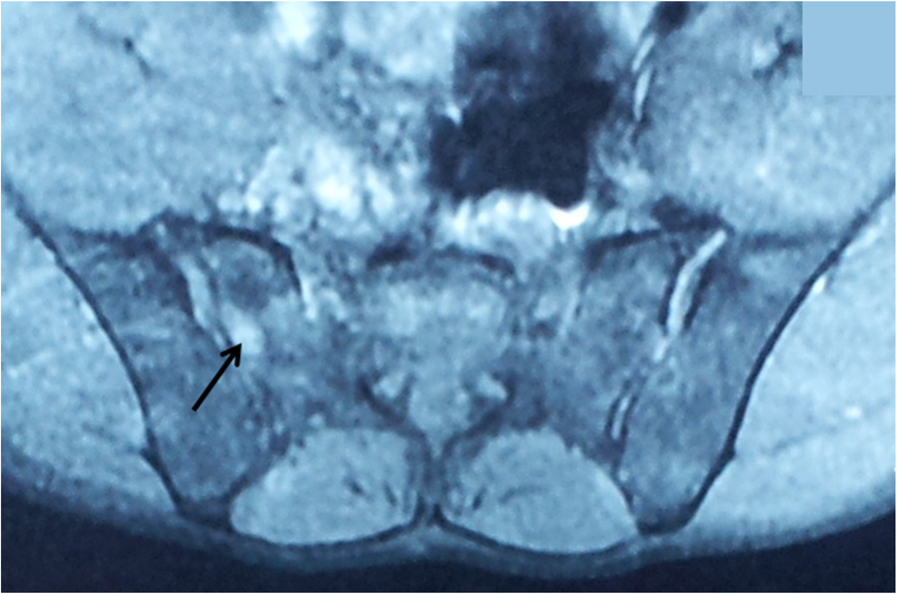
Magnetic resonance imaging of the sacroiliac joints showing bilateral sacroiliitis with a hyperintense signal on T2 (arrow) of the iliac and sacral banks, and sacroiliac spacing.

A 33-year-old Moroccan man with an unremarkable medical history, presented with large joints polyarthralgia, inflammatory pain in his buttocks and lower lumbar spine (morning stiffness of 45 minutes and frequent nocturnal awakenings), fever and skin lesions. He had no family history of rheumatic or skin diseases. On physical examination, the patient had a low-grade fever (38–39°C), six tender but not swollen joints (shoulders, elbows, and knees), a marked limitation of his lumbar spine flexion (Schober index=10+2cm, finger-to-ground distance=10cm), and painful erythematous maculopapules over his face, neck, and hands. Laboratory tests showed hyperleukocytosis (12,140/mm^3^ with 8500/mm^3^ of neutrophils), elevated erythrocyte sedimentation rate (106 mm/hour), and raised C-reactive protein (CRP) (93 mg/L). The results of the immunological tests including antinuclear antibodies, rheumatoid factor, and anti-cyclic citrullinated peptide antibodies were negative. The results of urine culture, blood culture, tuberculin skin test, search of *tubercle bacilli* in the sputum, and serology of cytomegalovirus and hepatitis were negative. Investigations for an underlying neoplastic disease remained negative. The patient’s chest X-ray was normal, whereas X-rays of the patient’s skeleton showed a squaring of the lumbar vertebrae (Figure 1), and suspected bilateral sacroiliitis that was confirmed on magnetic resonance imaging (MRI) of the sacroiliac joints (Figure 2). A skin biopsy revealed a neutrophilic infiltrate in the dermis and epidermis.

A diagnosis of SwS was based on two major and two minor criteria proposed by Su and Liu and modified by Von Den Driesch [6,7] (Table 1). The diagnosis of SwS associated with AS (the AS criteria of the Assessment of SpondyloArthritis international Society were fulfilled) was established. The patient received indomethacin (150 mg/day) and showed rapid onset of improvement. Within a few days, the progression of the skin lesions had stopped and they started to disappear. After one week, fever and pain disappeared with a decreasing CRP level (20 mg/L).

**Table 1:** **Criteria proposed by Su and Liu **[6]** for the diagnostic of Sweet’s syndrome**

**Major criteria**	**Minor criteria**
· Abrupt onset of typical cutaneous lesions.	· Fever < 38°C
· Histopathologic evidence of a dense neutrophilic infiltrate without evidence of leukocytoclastic vasculitis.	· Association with an underlying hematological disorder, malignancy, inflammatory disease, or pregnancy, or preceded by an upper respiratory or gastrointestinal infection or vaccination.
	· Abnormal laboratory values at presentation (3 of the following 4): Erythrocyte sedimentation rate greater than 20 mm/hour, positive C-reactive protein, greater than 8000 leukocytes, and greater than 70% neutrophils.
	· Good response to systemic corticosteroids, potassium iodide, or steroids.

The patient must fulfill two major and two minor criteria for the diagnosis.

## Discussion

SwS remains a rare condition that can be drug induced (lithium, furosemide, oral contraceptives, trimethoprim-sulfamethoxazole, and so on) or associated with other pathologies, including infections in 20 to 30% of cases (bacterial, viral infections), malignancies in 10 to 20% of cases (hematological disorders in 75% of cases especially acute myelogenous leukemia, solid tumors in 35%, such as carcinomas of the genitourinary organs, breast, thyroid, and gastrointestinal tract), and chronic inflammatory disorders in 10 to 20% of cases (rheumatoid arthritis, Behcet’s disease, inflammatory bowel disorders, sarcoidosis, Sjögren’s syndrome, and systemic lupus erythematous) [1,3,8,9]. However, the association with spondylarthropathy has rarely been described. Only three cases of SwS associated with this rheumatic disease have been reported [3-5]. In two cases, the patients presented with a long-standing association: AS fulfilling the New York criteria, and SwS according to Su and Liu’s criteria [6]. In one of these two cases, an acute Crohn’s colitis had developed. In the third case, SwS was concomitant with the AS as in our patient.

This association might be regarded as a mere coincidence; however, two facts raise some questions: SwS has already been reported to be associated with other inflammatory diseases, and the presence of three more cases reporting the association between SwS and spondylarthropathy.

No definite pathogenetic conclusions about the connection between SwS and AS can be drawn at the moment, because the respective pathogeneses of both diseases have not yet been clarified. Initial hypotheses have been raised regarding the deposition of immune complexes on the vessel walls, similar to the type III hypersensitivity reactions causing immune complex-mediated vasculitis. Other hypotheses, which have not been supported by experimental data, have been suggested including T-cell-dependent cellular immune activation, and altered neutrophil function [3,7]. It is currently thought that impairment of neutrophil function and cytokine dysregulation (interleukins, granulocyte colony-stimulating factor, interferon gamma, and so on) may represent a common link between AS and SwS [4,10]. An infectious hypothesis has also been suggested for the pathogenesis of spondylarthropathy and SwS: the same infectious agent could be the cause of these diseases [5]. Taking into consideration all the currently available data, the pathogenetic mechanisms responsible for the association between SwS and AS remain not well understood.

## Conclusions

Spondylarthropathy may be added to the list of musculoskeletal disorders associated with SwS. The rarity of such case reports argues for an association by chance and, accordingly, to the lack of relationship between SwS and this rheumatic disease. Nevertheless, there are hypotheses that have not been supported by experimental data, which can explain the mechanism responsible for such an association. The diagnosis of this neutrophilic dermatosis must seek systematically an associated pathology including spondylarthropathy.

## Consent

Written informed consent was obtained from the patient for publication of this case report and accompanying images. A copy of the written consent is available for review by the Editor-in-Chief of this journal.

## Abbreviations

AS: Ankylosing spondylitis; CRP: C-reactive protein; SwS: Sweet’s syndrome.

## Competing interests

The authors declare that they have no competing interests.

## Authors’ contributions

SM, FA, NA, AB, and ST analyzed and interpreted the patient data regarding the rheumatic and dermatological disease. MF, FA, LT, FM and TH interpreted the patient data regarding the rheumatic disease. All authors read and approved the final manuscript.
